# ASF1b is a novel prognostic predictor associated with cell cycle signaling pathway in gastric cancer

**DOI:** 10.7150/jca.69544

**Published:** 2022-03-28

**Authors:** Chuanzhi Chen, Haili Bao, Wu Lin, Xiangliu Chen, Yingying Huang, Haohao Wang, Yan Yang, Jin Liu, Xiadong Lv, Lisong Teng

**Affiliations:** 1 Department of Surgical Oncology, The First Affiliated Hospital, School of Medicine, Zhejiang University, Hangzhou 310003, China.; 2 Department of Organ Transplantation, Shanghai Changzheng Hospital, Navy Military Medical University, Shanghai, 200003, China.

**Keywords:** Gastric cancer, ASF1b, cell cycle, cell proliferation, prognosis, PI3K/AKT/mTOR signaling.

## Abstract

Gastric cancer (GC) is one of the most common malignant tumors with poor outcomes. Identification of new therapeutic targets is urgently needed. Accumulating evidence has shown that anti-silencing function 1b (ASF1b) contributes to the progression in multiple cancer types. However, detailed mechanisms of ASF1b tumorigenesis in gastric cancer remain elusive. This study showed that ASF1b was upregulated in GC tissues and remarkably correlated with TNM stage, histological grade and poor prognosis of GC. We induced down and up-regulation of ASF1b in GC cell lines and monitored the changes in their biological behavior. Furthermore, loss of ASF1b was efficient to suppress subcutaneous xenograft tumor growth *in vivo*. We demonstrate that ASF1b is involved in regulation of cell cycle and PI3K/AKT/mTOR signaling through experiments and database analysis. Mechanistically, ASF1b promoted the proliferation, migration and invasion of GC cells. Taken together, this study highlights the role of ASF1b, which provided new insights into the underlying mechanism of progression and metastasis in GC for the first time.

## Introduction

Gastric cancer (GC) is one of the most common malignancy with high cancer mortality rates worldwide. According to the International Agency for Research on Cancer (IARC) GLOBOCAN cancer statistics in 2020. There were 5.6% gastric cancer in all new cancer cases diagnosed. In addition, GC was the fifth most commonly diagnosed cancer type which responsible for 7.7% of all deaths from cancer after lung cancer (18.0%), colorectal cancer (9.4%), liver cancer (8.3%) 1-3. Remarkably, in east Asia, China almost had half of the global incident cases in 2017 with 562 thousand 4. With an increasing burden of GC incidence, further investigation on the epidemiology, mechanisms and treatment of GC is urgently needed 5. It is well-established that GC is an intertumoral heterogeneous and genotypic disease, the occurrence and progression of GC is closely associated with gene expression profiles. Although clinical GC treatments, including neoadjuvant chemotherapy, radical surgery and conventional radiation therapy are usually performed, patient-specific still leads to ineffective treatment and poor clinical outcomes 6.

Currently, precise classification and new therapies have been widely applied in GC. With the rapid development of molecular typing and molecular diagnosis in cancer, the underlying mechanism of GC were explored in the help of big data bioinformatics, Next-generation sequencing (NGS) and drug screening in xenograft model 7, 8. Expression profile of markers such as Human epidermal growth factor receptor-2 (HER2), microsatellite instability (MSI) and PD-L1 was approved for targeted therapy in gastric cancer patients 9. Furthermore, Bcl-2, CCND1, MUC, MSI, CD10 and so on were regarded as prognostic markers which influence the treatment strategies for GC patient 10-14. Targeted therapies include trastuzumab for HER2-positive patients and nivolumab or pembrolizumab for anti-PD-1 were licensed to treat GC 15. To improve the prognosis of GC, new insights into the occurrence and mechanisms of formation still need further investigation.

Gene Expression Profiling Interactive Analysis (GEPIA) was proved to be an effective integrated data-mining platform for identify differential expression genes (DEGs) by investigating the transcriptional and survival data 16. As more than 90% of all GC are stomach adenocarcinomas (STAD), comparison of the gene-expression profiles of the STAD and normal samples to excavate the DEGs and novel potential prognostic marker for GC patients 17.

It is well-established that abnormal gene expression, teaming up alterations in DNA methylation and histone modifications, including gene overexpression or silencing 18. Structural organization of chromatin with tumor suppressor properties has been shown to be involved in the occurrence and progression of human tumor, including histone modifying enzymes, histone chaperone proteins, binding/effector proteins and ATP‐dependent nucleosome remodeling proteins 19, 20. Anti-silencing function 1 (ASF1) is a conserved H3-H4 histone chaperone that plays a role in DNA replication and repair, and transcriptional regulation 21. ASF1b, the homologs to ASF1, has been found involved in cellular proliferation and cellular death 22. Interestingly, previous study revealed that an increased ASF1b mRNA level was correlated with clinicopathological features and disease outcome in cervical cancer, and the researchers suggested that ASF1b might an independent prognostic biomarker and a novel therapeutic target in cervical cancer patients 23. In lung adenocarcinoma (LUAD), knocking down ASF1b impaired the proliferation and induced cell apoptosis 24. Recent research also noted that ASF1b may serve as a prognostic biomarker associated with immunotherapy for several cancers 25. However, the distinct expression and function of ASF1b in GC is not clear.

In our research, ASF1b was identified as a potential candidate oncogene in GC through analyzing data collected from both public databases and patient samples in GC. Moreover, we induced ASF1b overexpression and knockdown in GC cell lines and conducted a comprehensive study *in vitro* to investigate the biological behavior change. Furthermore, BALB/C nude models were established using stable AAV-shRNA-ASF1b AGS cells or AAV-shRNA vector AGS cells to assess how ASF1b knockdown suppresses tumor growth. Our results show that ASF1b was closely associated with proliferation, migration and angiogenesis in GC cells and might be an independent prognostic marker and potential therapeutic target for GC.

## Materials and methods

### Patients and tissue samples

According to the histopathological results, we randomly collected 200 GC samples from the First Affiliated Hospital of Zhejiang University during February 2012 to December 2016. All the patients enrolled were non-gastroesophageal junction tumor and had not received adjuvant chemotherapy treatment or radiation therapy prior to surgery. These patients had available data, including clinicopathological features and follow-up information through inpatient chart review and telephone calls. Exclusion criteria included previous GC surgery, insulin-requiring diabetes, severe heart failure, renal and hepatic failure or dysfunction in cirrhotic. Tumor staging of the GC patients was classified according to the American Joint Committee on Cancer 8th edition (AJCC 8th edition) criteria. The survival of patients was tracked until July 2021. We developed Quantitative PCR (qPCR) of 40 GC tissue samples and matched normal adjacent tissues (NAT) to evaluating ASF1b expression in GC samples. High-throughput tissue microarray (TMA) sets for immunohistochemistry (IHC) included 160 paired GC tissue samples were applied to investigate the correlation between ASF1b and clinicopathological features. Both the univariate and multivariate Cox analyses were carried out to evaluate the prognostic significance of the ASF1b expression in overall survival (OS) and disease-free survival (DFS). This study was approved by the local hospital ethics committee (Ethical approval No. 2021433), and all patients involved in this study have written informed consent prior to enrollment.

### Cell lines and cell culture

Human GC cell lines, including HGC-27, AGS, MGC-803, MKN-45, SNU-1, GES-1 (normal gastric cell line) and human embryonic kidney cell line (HEK293T) were purchased from the Cell Bank of the Chinese Academy of Sciences (Shanghai, China). HGC-27, AGS, MGC-803, MKN-45 and SNU-1 were cultured in RPMI 1640 (Gibco, Rockville, MD, USA) supplemented with 10% fetal bovine serum (FBS) (Gibco, Rockville, MD, USA) and 1% antibiotics. GES-1 and HEK293T were cultured in DMEM (Gibco, Rockville, MD, USA) supplemented with 10% FBS and 1% antibiotics. Cells were digested by 0.25% Trypsin/EDTA (Gibco, Waltham, MA, USA) in logarithmic growth phase. All of the cells were cultured at 37 °C in a fully humidified atmosphere of 5 % CO2.

### RNA Extraction and qPCR

According to the manufacturer's instructions, the total RNA from the tissue samples and cells was extracted by TRIzol reagent (Invitrogen, Carlsbad, California, USA). Then, the cDNA was synthesized with a PrimeScript RT reagent kit (Takara, Kyoto, Japan). Real-time qPCR analyses were performed with SYBR Green reaction system (Takara, Kyoto, Japan). The relative expression of ASF1b was quantified using the 2-ΔΔCT method, with GAPDH as the internal control. The specific primers sequences used were listed in Table I.

### Small Interfering RNA (siRNA) and overexpression plasmids

Negative control siRNA (NC) and siRNA-targeting the human ASF1b (ASF1b-si) sequence were purchased from GenePharma company (Shanghai, China). PcDNA3.1 with ASF1b overexpression (ASF1b-OE) and empty pcDNA3.1 (vector) were provided by GENEray. Cells were transfected with Lipofectamine 3000 transfection reagent (Invitrogen, Carlsbad, California, USA) in Opti-MEM medium (Gibco, Carlsbad, California, USA). For each 6-well plate, 5 nM siRNA and 400ng plasmid were diluted in 200 μL opti-MEM medium respectively. Then, added the mixture of transfection chemicals and Lipofectamine 3000 reagent into individual well for 6 h before replacing with fresh complete medium in accordance with the manufacturer's instructions.

### AAV-shRNA-ASF1b construction and AAV virus packaging

The AAV-shRNA-ASF1b plasmid (pCDH-CMV-MCS-EF1-CopGFP-T2A-puro) was constructed by GENEray (Shanghai, China). HEK293T cells were transfected with a plasmid mix of short hairpin RNA constructs targeting ASF1b, pMD2.G and psPAX2 using Opti-MEM medium and Lipofectamine 3000. ShRNA sequences were shown in Table I. The culture supernatants were collected at 48 h post transfection, centrifuged, and stored at -80 °C. Subsequently the viral supernatants were incubated with GC cells for 48 h in a 6-well plate at 37 °C in incubator. Stable ASF1b knockdown GC cells were selected using puromycin (2 μg/ml) for 7 d, and the ASF1b protein expression level was identified through Western blot and qPCR.

### IHC staining

IHC was performed both on TMAs sections and mouse tumor sections. Paraffin-embedded sections were deparaffinized in xylene, rehydrated through graded alcohols and incubated with 3% hydrogen peroxide for 10 min. The slides were then immunostained with primary antibodies for ASF1b (1:100, Santa Cruz Biotechnologies), vascular endothelial growth factor (VEGF) (1:100, Cell Signaling Technology) or Ki67 (1:100, Cell Signaling Technologies) at 4◦ C overnight, and incubated with secondary antibody (ZSGB- bio, Beijing, China) at room temperature for 30 min. Then the slides were stained with DAB Chromogen (ZSGB-bio, Beijing, China) and counterstained with hematoxylin. ASF1b expression was evaluated based on the percentage of positive-stained area. The percentage of the staining was categorized as 1 (0-25%); 2 (26-50%); 3 (51-75%); 4 (>75%), and the staining intensity was graded on a scale of 0 to 3, with 0 (negative); 1 (weak); 2 (moderate) or 3 (strong). IHC score were evaluated by multiplying the frequency and intensity scores.

### Cell viability assay

Cell viability assay was examined using the Cell Counting Kit-8 (CCK-8; APExBIO, Houston, USA) according to the manufacturer's instructions. Briefly, 2,000 cells were seeded into 96-well plates. after 0h, 24h, 48h, and 72h post-transfection, 10 μl CCK-8 solution in 100 μl complete medium was added to each well. After that, the cells were incubated for 2 h at 37 °C. Then, the absorbance was measured at 450 nm wavelength.

### Colony formation assay

Cells were harvested during exponential growth. Briefly, 1000 cells per well were inoculated in 6-well plates and incubated for 10-14 days in RPMI-1640 medium. The colonies were stained with a 0.05% crystal violet solution after washing with PBS. Images of colonies were captured and subsequently counted with ImageJ (version1.53a; National Institutes of Health, USA).

### Cell Migration and Invasion Assays

The tumor cells (3.0 × 10^4^) were seeded into 24-well format transwell chambers with 8.0um pore polycarbonate filter inserts (Costar, Cambridge, MA, USA). The chambers were placed in 24-well plates containing RPMI-1640 medium and 10% FBS. For migration assays, cells were suspended in 200 μl serum-free RPMI 1640 medium and cultured in the upper chamber. The lower chambers were filled with 700 μl RPMI-1640 and 10% FBS. For invasion assays, the inserts were coated membranes with Matrigel (50 μl/well) (BD Biosciences, Lake Franklin, NJ, USA) before adding the cells. Cells were incubated at 37°C for 24 h. Subsequently, filters were fixed using methanol for 15 min, stained with 0.1% crystal violet for 15 min, and washed twice with PBS. The number of cells were counted in five randomly selected microscopic views (100x).

### Protein extraction and Western blotting analysis

Cells were washed twice using PBS, then total protein was extracted using Radio-Immune Precipitation Assay (RIPA) Lysis Buffer (Sigma, MO, USA) mixed with phenylmethanesulfonyl fluoride (PMSF) (Bioship, Anhui, China) and serine-type phosphatase inhibitor (Servicebio, Wuhan, China). The protein concentration was examined using the BCA Protein Assay Kit (Thermo Fisher Scientific, Massachusetts, USA). 20 μg per sample were separated by SDS-PAGE and transferred to PVDF membrane. Membranes were blocked with 10% skim milk for 1 h at room temperature and incubated overnight with primary antibodies at 4 °C. After washed thrice with TBST (10 min each), membranes were incubated with an appropriate secondary antibody at room temperature for 1-2 h. After washed secondary antibody, membranes were imaged via enhanced chemiluminescence (ECL) solution (Bio-red, California, USA). The quantitative changes in the luminescence were estimated by ImageJ.

The primary antibodies are as follows: cyclin-dependent kinase (CDK2, # ab101682), CDK4 (#ab137675), cyclin D1 (#ab16663), P21 (#ab109520), P27 (#ab190851), mTOR (#ab32028), p-mTOR (#ab109268), PI3K (#ab191606) (Abcam, Cambridge, UK); p-PI3K (#17366), AKT (#4691), p-AKT (#4060) (CST, Boston, USA); ASF1b (#sc-393169, Santa Cruz, California, USA) and GAPDH (#60004-1-Ig, Proteintech, Wuhan, China). These antibodies were diluted 1000-fold with primary antibody diluent (Fdbio science, Hangzhou, China).

### Flow cytometry and cell cycle assay

To perform cell cycle assays, cells (2 × 10^5^ - 1× 10^6^) were collected and then washed with PBS for thrice. Cell Cycle Staining Kit was purchased from Multi Sciences (Hangzhou, China). 1 ml DNA staining solution and 10ul Permeabilization solution was added and mixed. Incubated for 30 min at room temperature avoiding light away from light. Finally, cells were analyzed on FACSCalibur (BD Bioscience) and different phase of the cell cycle were conducted by FlowJo software.

### Bioinformatics Analyses

The DEGs in STAD were firstly explored from the GEPIA database (http://gepia2.cancer-pku.cn/#index) with p-value < 0.01 and | log2 fold changes | > 2 as the thresholds. DEGs was further screened by univariate Cox regression analysis with significant Hazard rate (HR) from OS and DFS (all, P < 0.05 log-rank test). We used Kaplan-Meier plotter (KM plotter) (http://kmplot.com/analysis/), which included the GEO, European Genome-phenome Archive (EGA), TCGA database, to investigate the relationship between ASF1b expression and clinicopathological features in STAD, and further verified via Oncomine (www.oncomine.org). Co-expression genes (CEGs) of ASF1b were predicted by cBioPortal (http://www.cbioportal.org) online analysis with |Spearman's r| ≥ 0.5 as the threshold. In addition, Gene Oncology (GO) terms enrichment analysis of the CEGs were elucidated with David 6.8 database, including biological processes (BP), molecular function (MF) and cellular component (CC). Meanwhile, Kyoto Encyclopedia of Genes and Genomes (KEGG) pathways enrichment was performed at KOBAS 3.0 (http://kobas.cbi.pku.edu.cn/).

### Subcutaneous tumor growth *in vivo*

Female BALB/c nude mice were purchased from Hangzhou Ziyuan Laboratory Animal Technology Co., Ltd. (Hangzhou, China). To evaluate the relevance of ASF1b expression to tumor growth *in vivo*, AGS cells (5×10^6^ cells in 100 μl PBS per mouse) stably transfected with shRNA-NC and shRNA-ASF1b were subcutaneously inoculated into left flanks of mice (5 weeks, n = 5 every group), respectively. Tumor growth was monitored for every week. At 4 weeks after injection, mice were sacrificed, tumors were isolated from the sacrificed mice, and fixed in formalin. All animal work was performed at the First Affiliated Hospital of Zhejiang University. Animal experiments were approved by the Ethics Committee of the First Affiliated Hospital, College of Medicine, Zhejiang University (Ethical approval No. 2021-924), and conducted according to the Guidelines for Care and Use of Laboratory Animals of Zhejiang University.

### Statistical Analysis

The differences between gastric cancer tissues and paired adjacent normal tissues were assessed using a two-tailed paired Student t test. T test was used for comparison between two groups while one-way analysis of variance (ANOVA) was used to compare differences among multiple groups. The correlations between ASF1b expression and the relevant clinicopathological features of GC patients were analyzed using the Pearson χ2 test and Fisher's exact test in SPSS. Kaplan-Meier survival curves was constructed using the “Survival” package in R. The univariate and multivariate Cox proportional hazards multiple regression model was performed by “forestplot” package to investigate the prognostic significance. Statistical analysis was performed with GraphPad Prism 9 software, SPSS 26.0 software and R 4.0.4 program. A p value of < 0.05 was considered statistically significant. All cell experiments were repeated three times independently.

## Results

### ASF1b is a potential oncogene in GC

**Figure 1:**
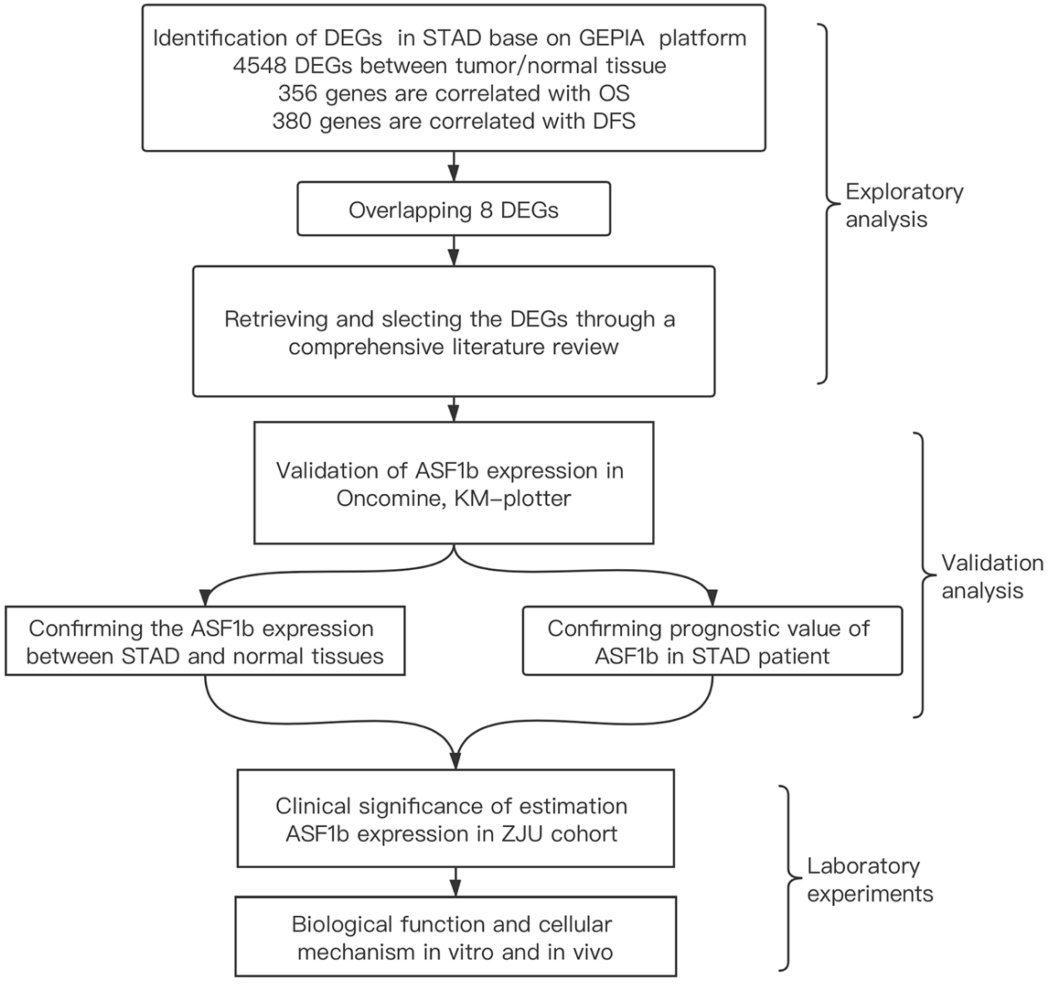
The integrative analytic strategy in this study. Stomach adenocarcinomas (STAD), Differential expression genes (DEGs), Kaplan-Meier Plotter database (KM-Plotter), Zhejiang University (ZJU).

We identified the ASF1b as a potential biomarker in STAD though a multi-step analytical strategy (Figure 1). The figure elaborates our study's guiding framework, which divides the process into three constituent parts: (1) exploring the potential biomarkers for STAD on GEPIA and literature review; (2) verify ASF1b expression and its clinical prognostic value in STAD using Oncomine and Kaplan-Meier plotter (3) confirm ASF1b both on human genetic data and functional assays (*in vitro* and *in vivo*). Base on GEPIA database, we excavated 4548 DEGs between tumor and normal tissue in STAD including over-expressed and under-expressed. Furthermore, survival analysis shows 356 and 380 genes were significantly correlated with OS and DFS, respectively. Finally, we computed the intersection of the three gene sets to obtain 8 genes (Table S1 and Figure S1F) potentially involved in the progression of STAD. After reviewing relevant literature and assessing potential scientific value of the genes, we ultimately selected ASF1b for the subsequent studies and experiments.

### ASF1b expression was upregulated in GC and stable cell lines

**Figure 2:**
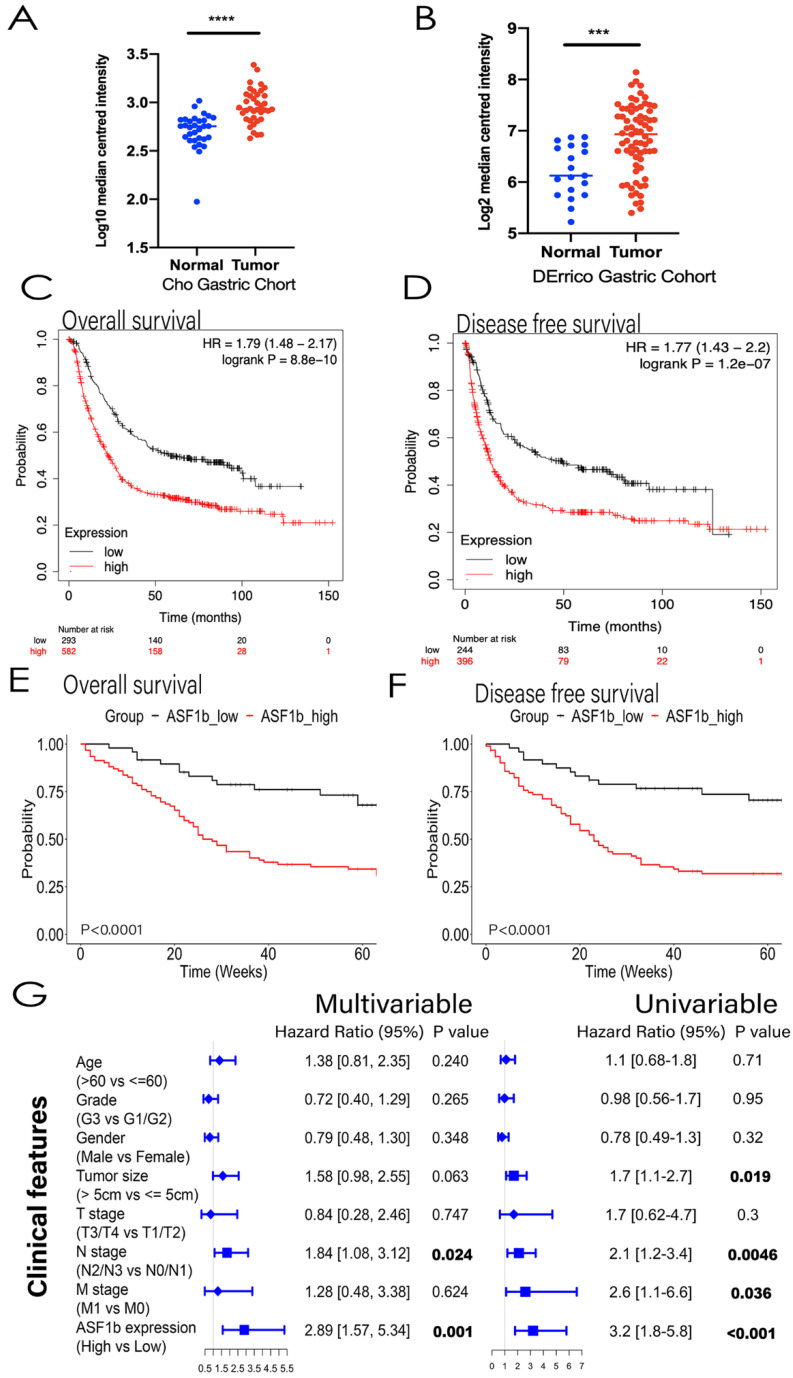
(A,B) ASF1b mRNA levels of GC vs. normal adjacent tissues (NAT) in Oncomine database (Cho Gastric, DErrico Gastric ,P<0.0001). (C) GEPIA revealed ASF1b was significantly upregulated in 408 GC tissues. (D) ASF1b was significantly upregulated at most of tumors except Acute Myeloid Leukemia (LAML) and Testicular Germ Cell Tumors (TGCT) (E,F) The association between ASF1b expression and overall survival (OS), disease-free survival (DFS) in GC patients assessed by K-M plotter, respectively. (G,H) The association between ASF1b expression and OS, DFS in GC patients assessed by ZJU cohort, respectively. (I) Multivariable and univariable analyses were performed in Zhejiang cohort. All the bars correspond to 95% confidence intervals.

**Figure 3:**
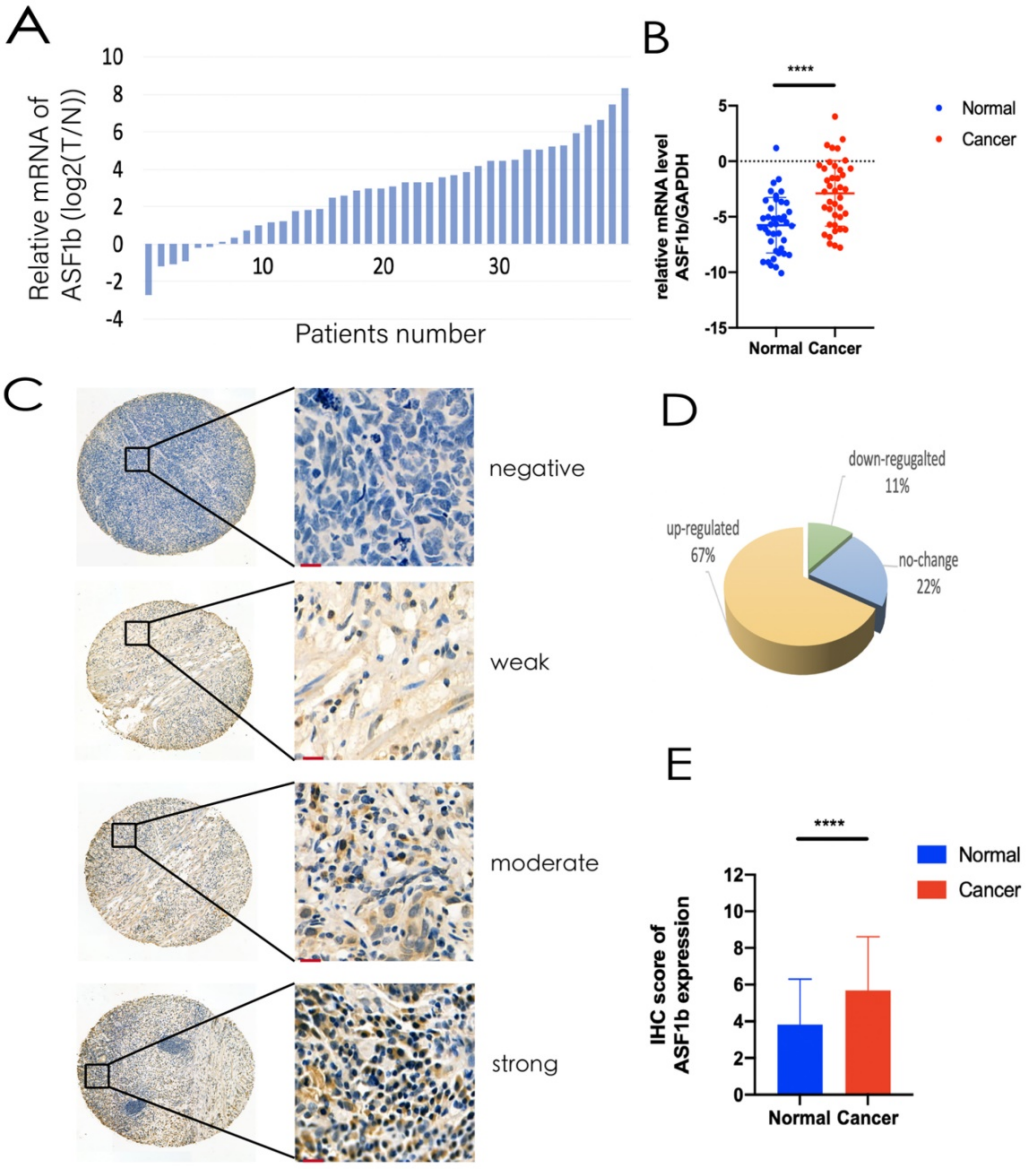
(A,B) ASF1b mRNA levels of GC vs. NAT in ZJU cohort. (B) Relative expression of ASF1b mRNA in GC tissues and their corresponding NAT was determined by qRT-PCR and expressed as -ΔΔCT (****P<0.0001). (C) Representative immunohistochemical staining of ASF1b in GC tissues and NAT (scale bar = 10 um). (D) A GC tissue microarray (TMA) (n = 160) indicated that the expression of ASF1b was upregulated in 65% of GC tissues. (E) The protein expression levels of ASF1b in 160 paired TMA (****P<0.0001).

Analysis of GEPIA data revealed ASF1b was significantly upregulated in 408 GC tissues compared to the 211 normal gastric tissues (Figure S1D). Same result was found almost at all kinds of tumors except Acute Myeloid Leukemia (LAML) and Testicular Germ Cell Tumors (TGCT) (Figure S1E). Similarly, high ASF1b expression states was observed in the Oncomine Cho Gastric and DErrico Gastric tumor samples (Figure 2A and 2B, P<0.001). We measured the expression levels of ASF1b mRNA in 40 pairs of primary GC tissues and NAT on ZJU cohort. Consistent with the TCGA databases (GEPIA and Oncomine), the expression of ASF1b mRNA was significantly higher in GC tissues (Figure 3A and 3B). Moreover, we analyzed the ASF1b protein expression on TMA in 160 GC cases using IHC score (Figure 3C), which confirmed that the expression of ASF1b was upregulated compare to NAT in 67% of GC tissues (Figure 3D and 3E).

**Figure 4:**
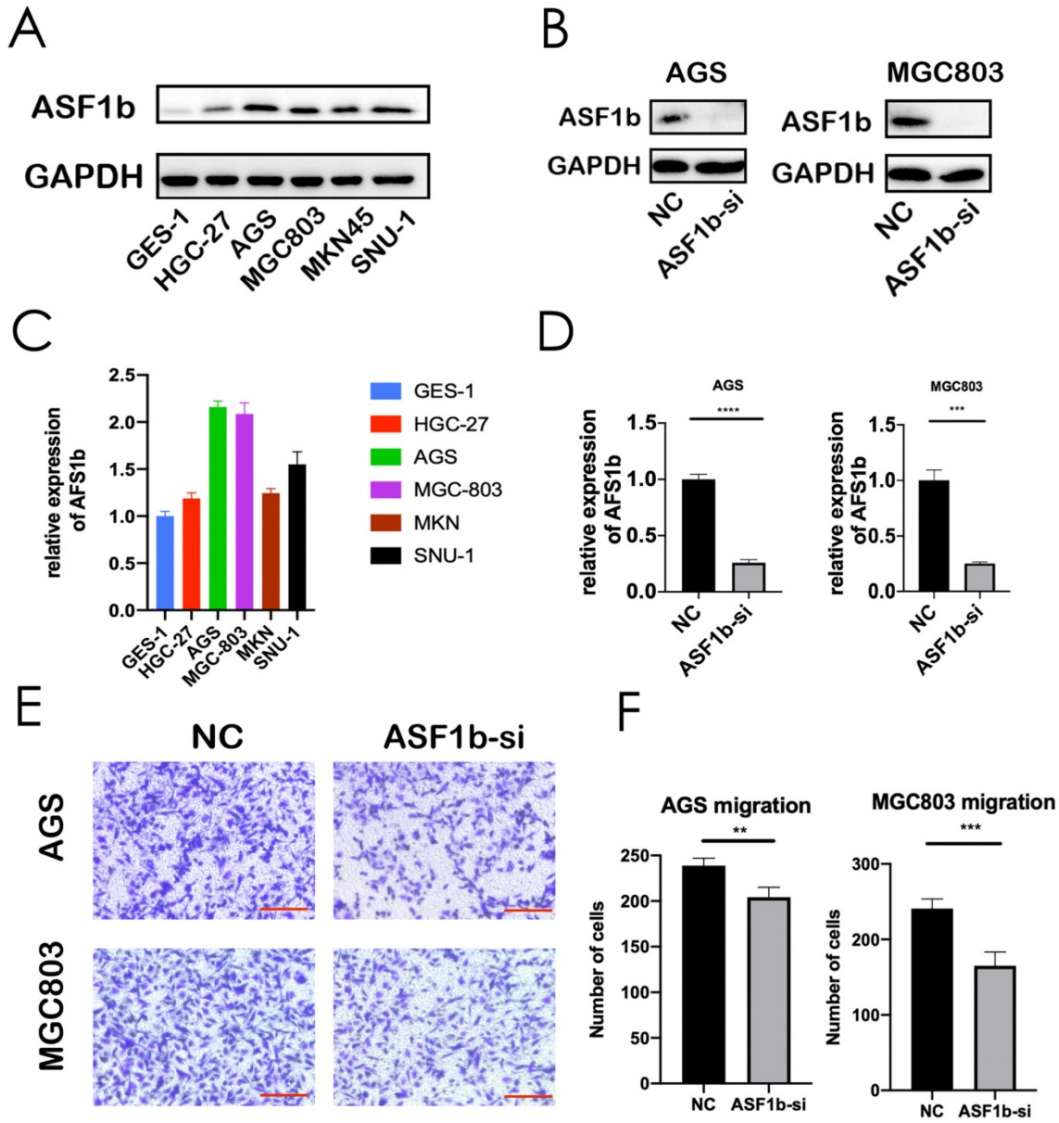
(A,C) Protein expression and mRNA levels of ASF1b were checked in a panel of normal gastric cells (GES-1) and five human GC cell lines. (B,D) Protein expression and mRNA level of ASF1b were efficiently inhibited by ASF1b-si in AGS and MGC803 cells. (E,F) Migration assay was performed in AGS and MGC803 cells transfected with ASF1b-si/NC (scale bar = 20 μm, **P < 0.01; ***P < 0.001).

Furthermore, we investigated whether ASF1b expression was elevated in six different GC cell lines: HGC27, AGS, MGC803, MKN45, SNU1 and normal gastric cell line (GES-1). Western blot assay was repeated three times independently, and the mean gray levels of the protein bands were quantified by using the ImageJ program (Figure S1C). Western blot results indicated that ASF1b protein expression level was much higher in GC cells than in GES-1 cells (Figure 4A, Figure S1A and B). Moreover, the qPCR analysis of the cell lines further showed that ASF1b mRNA levels were increased in the five GC cell lines (Figure 4C). Overall, these data suggested that ASF1b was aberrantly upregulated in GC.

### GC Patients' characteristics with ASF1b

Kaplan-Meier curves for ASF1b mRNA expression level and prognostic outcome in GC, using KM plotter (http://kmplot.com/analysis) database, showed that high ASF1b was significantly associated with poor OS and DFS in GC patients (Figure 2C and 2D). Furthermore, we collected and analyzed the association between ASF1b protein expression and clinicopathological features in 160 GC cases via IHC score. As shown in Table II, high ASF1b expression (IHC scores ≥ 6) was significantly correlated with aggressive clinicopathological features, including high grade (p=0.009) and advanced clinical stage (p=0.007). Furthermore, we sought to validate the key role of ASF1b as a prognostic factor at the protein level. Therefore, all the patients who had complete clinicopathological data were followed up in ZJU cohort. As a result, a total of 20 patients (12.5%) were lost to follow-up. The remaining 140 patients were divided into two groups: ASF1b high-expression group (63.6%) and ASF1b low-expression group (36.4%). Survival curve comparison showed high-expression group had significantly worse OS and DFS (Figure 2E and 2F). In addition, univariate analyses revealed that tumor size (>5 cm), N stage (N2/N3), M stage (M1) and ASF1b expression (High) were independently associated with poor OS. Cox multivariate analysis further established N stage (N2/N3, HR=1.84) and ASF1b expression (High, HR=2.89) as prognostic factors for OS (Figure 2G). Collectively, these data suggested that ASF1b was associated with poor prognosis in GC.

### ASF1b Regulates proliferation, migration and invasion of Human GC Cell Lines

**Figure 5:**
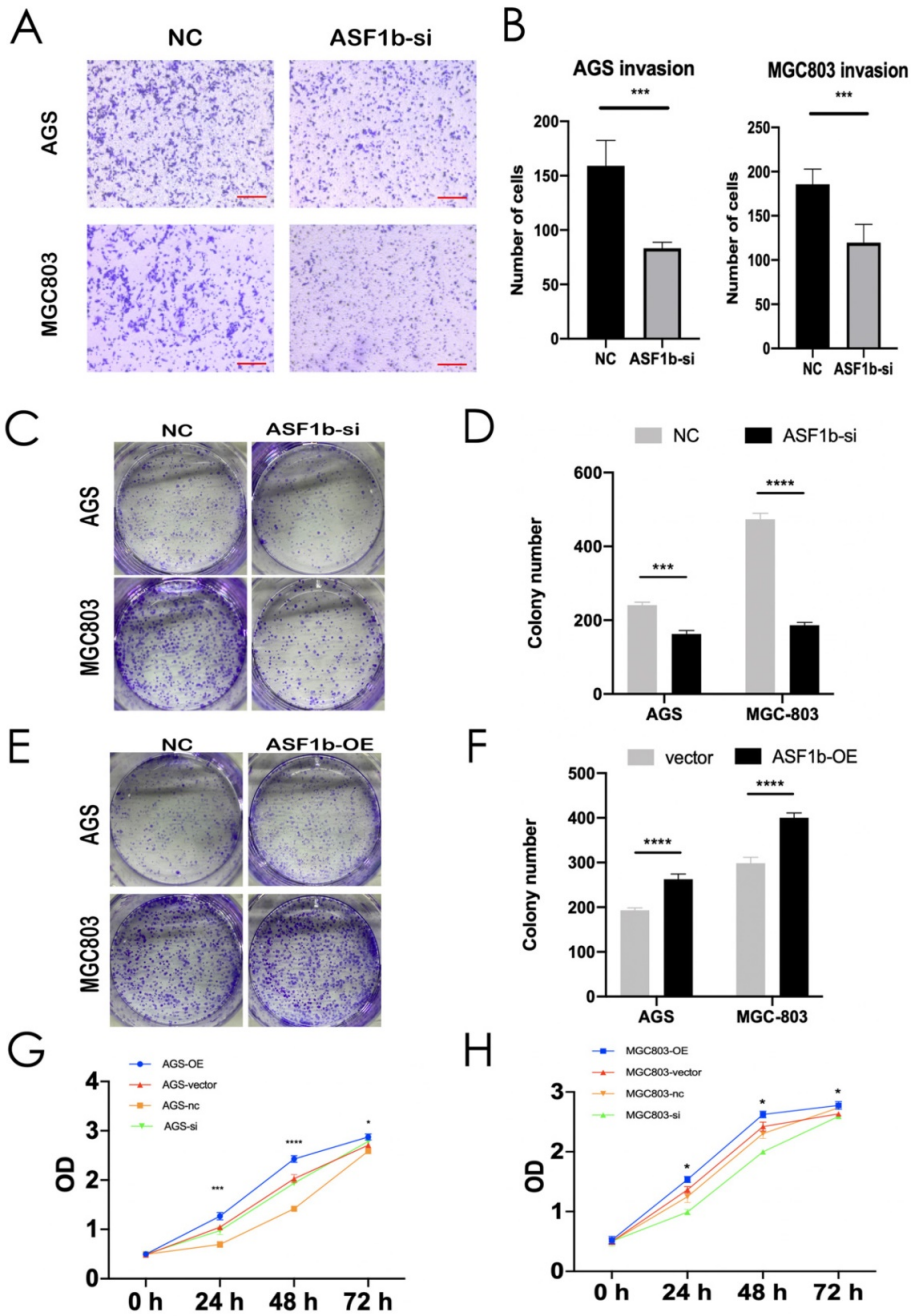
(A,B) Invasion assay was performed in AGS and MGC803 cells transfected with NC/ASF1b-si (scale bar = 20 μm, ***P < 0.001). (C,D) Colony formation assay in AGS and MGC803 cells transfected with NC/ASF1b-si. (E,F) Colony formation assay in AGS and MGC803 cells transfected with vector and ASF1b-overexpression (ASF1b-OE). (G) CCK-8 assay in AGS cells transfected with ASF1b-si/NC or vector/ASF1b-OE. (H) CCK-8 assay in MGC803 cells transfected with ASF1b-si/NC or vector/ASF1b-OE.

We successfully transfected si-NC and ASF1b-si into AGS cells and MGC803 cells. The protein and mRNA were detected by western blot and qPCR (Figure 4B and D). Similarly, to visualize the behavior of ASF1b upon overexpression, we constructed ASF1b overexpression cells by recombinant plasmid both in AGS and MGC803 cells (Figure S2A and B). To identify the effect of ASF1b on the proliferation of AGS and MGC803 cells, cell viability was examined using a CCK-8 kit. The results demonstrated that the viabilities were significantly attenuated in ASF1b-si transfected AGS and MGC803 cells compared with the NC. Conversely, the cells carrying the ASF1b-OE plasmids grew faster than that of the plasmid-vector cells (Figure 5G and H). In addition, to evaluate the effect of ASF1b on GC cell growth, colony formation analysis was also performed in the ASF1b overexpression and knockdown cells, which returned parallel results (Figure 5C-F). Transwell migration assay and transwell matrigel invasion assay demonstrated ASF1b expression play a vital role in migration and invasion of GC cells. AGS cells and MGC-803 cells which knockdown ASF1b via siRNA above, significantly prevented cell migration (Figure 4E and F) and invasion (Figure 5A and B). Moreover, we analyzed the effect of ASF1b overexpression on the motility of the AGS cells and MGC-803 cells. Results show ASF1b could promote migration (Figure S2C and D) and invasion (Figure S2E and F) abilities of GC cells. Taken together, these data revealed that ASF1b expression promoted the proliferation, migration and invasion of gastric cancer cells *in vitro*.

### ASF1b is involved in cell cycle and PI3K/AKT/mTOR pathway in GC

**Figure 6:**
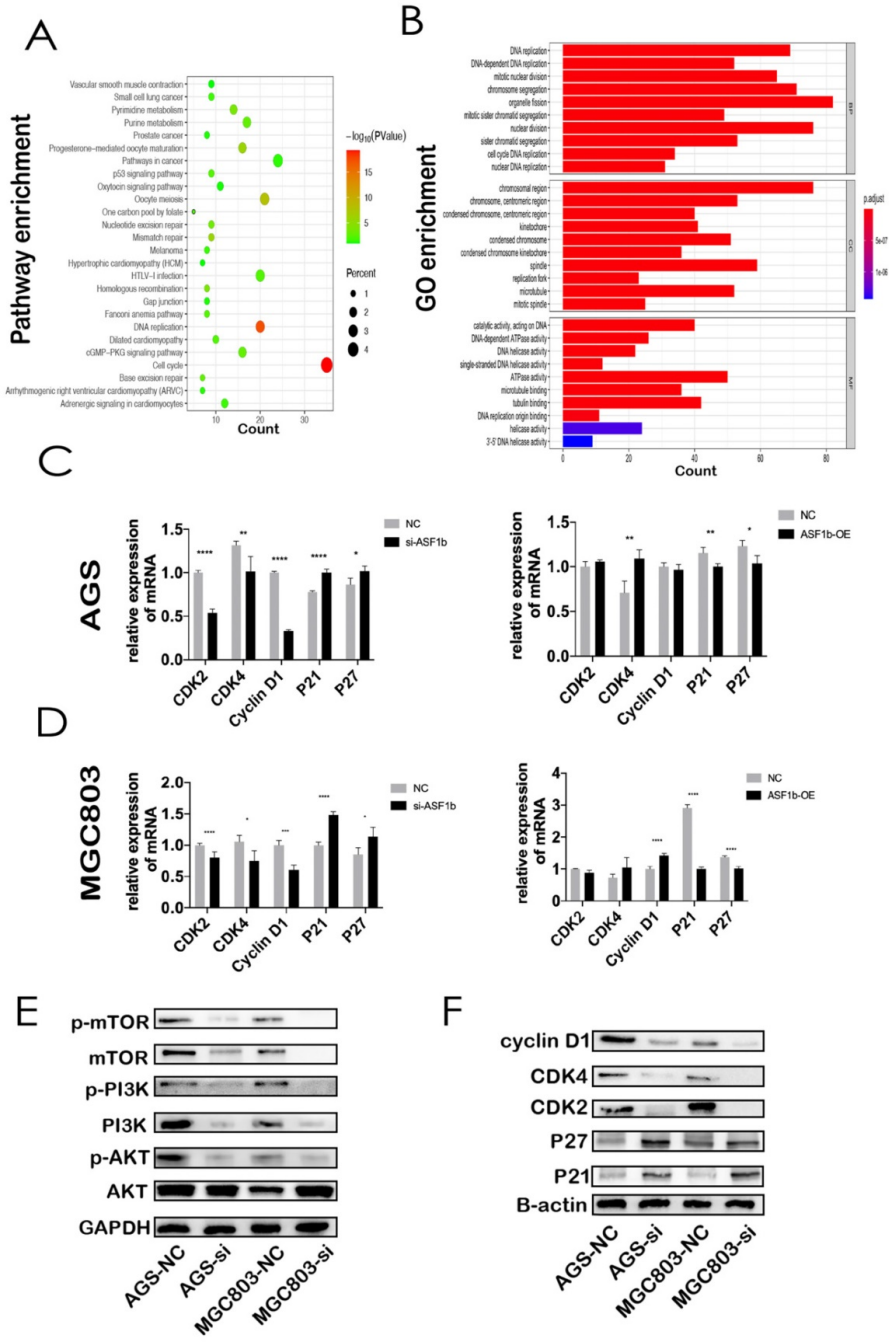
(A) KEGG pathway analysis of the genes significantly correlated with the ASF1b expression in GC from cBioPortal. Kyoto Encyclopedia of Genes and Genomes (KEGG). (B) GO analysis of ASF1b co-expressed genes in GC, Gene ontology (GO), Biological process(BP), Cellular component (CC), Molecular factor(MF). (C) MRNA levels of cell cycle-related markers in AGS cells transfected with ASF1b-si/NC using q-PCR. (D) MRNA levels of cell cycle-related markers in AGS cells transfected with vector/ASF1b-OE using q-PCR. (E) The protein expression levels of PI3K/AKT/mTOR pathway in GC cell lines after ASF1b-si. (F) The protein expression levels of cell cycle-related markers in GC cell lines after ASF1b-OE.

**Figure 7:**
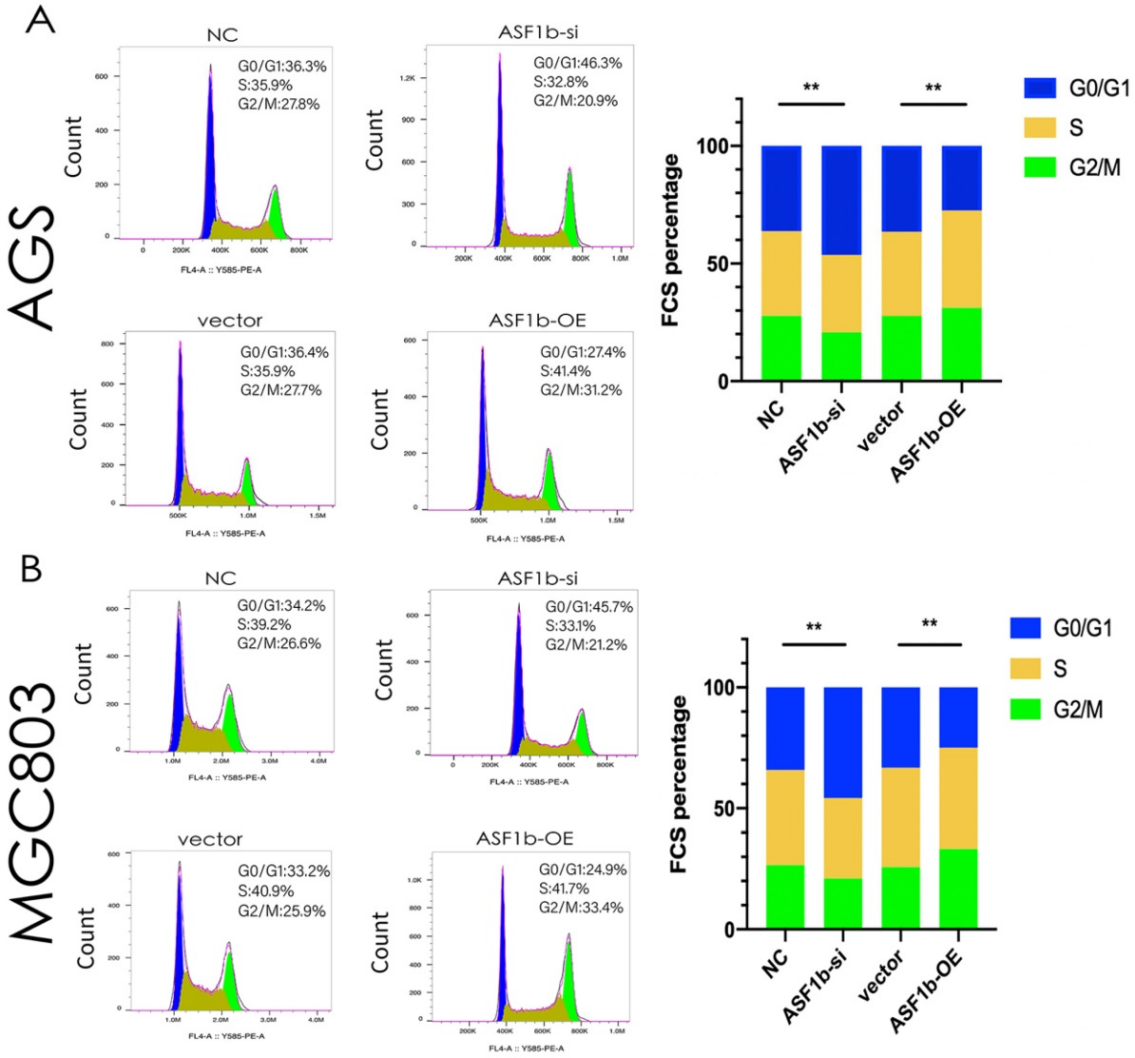
(A) Flow cytometry showing the percentages of AGS cells transfected with ASF1b-si/NC or vector/ASF1b-OE at different cell cycle phases. (B) Flow cytometry showing the percentages of MGC803 cells transfected with ASF1b-si/NC or vector/ASF1b-OE at different cell cycle phases.

Through co-expression analysis by cBioPortal database, we identified seven hundred and fifty-one genes co-expressed with ASF1b in GC (Table S1). KEGG analyses demonstrated that co-expressed gene-set of ASF1b was enriched in cell cycle pathways (Figure 6A). Meanwhile, GO functional enrichment analyses of CEGs support the idea that ASF1b was related to mitotic cell cycle processes, including organelle fission, chromosomal region, ATPase activity, suggesting that these CEGs could act as oncogenes to promote Human GC by accelerating cell cycle phase (Figure 6B). To precisely assess the cell cycle arrest induced by ASF1b, we monitored the cell cycle distribution after ASF1b-si and ASF1b-OE in AGS cells. Treatment with ASF1b-si induced arrest at the G1-S phase, accompanied by a decrease in the percentage of AGS cells in S phase and G2/M phase. On the contrary, ASF1b-OE reduced the cell cycle length by promoting the transition of AGS cells from G1 to S phase, which shortened the G0/G1 phase and accelerated cell cycle (Figure 7A). To confirm that ASF1b promoted Human GC cell cycle transition by shortening G0/G1, the same type of experiment was replicated in MGC803 cells. Similarly, ASF1b-si increased the proportion of G0/G1 phase cells and decreases the proportion of G2/M phase while ASF1b-OE induce opposite effect (Figure 7B).

Then, the expression of cell cycle-related proteins was measured to elucidate the specific role of ASF1b in proliferation. The expression of Cyclin D1, CDK2, CDK4, P21, P27 protein in ASF1b-si cells was determined by western blotting. The results showed that the suppression of ASF1b expression was associated with significant reduction of cyclin D1, CDK2, CDK4 expression. However, CDK inhibitor proteins P21 and P27 were markedly upregulated, suggesting that the induction of P21 and P27 enhanced the antiproliferative effect of ASF1b-si in GC cells (Figure 6F). Moreover, the corresponding mRNA levels of cell cycle-related markers were detected by q-PCR. Similarly, CDK2, CDK4 and Cyclin D1 displayed an opposite expression pattern to P21 and P27 both in AGS and MGC803 cells. It is worth noting that ASF1b-OE repressed the mRNA expression of P21 and P27 but did not significantly increased the CDK2, CDK4 and Cyclin D1 mRNA (Figure 6C and 6D). Additionally, we found levels of p-PI3K, p-AKT and p-mTOR were decreased in ASF1b-si AGS and MGC803 cells, demonstrating that ASF1b-si could markedly inhibited the PI3K/AKT/mTOR pathway (Figure 6E). Our data suggest that ASF1b was a putative proliferation-associated marker in Human GC.

### Knockdown of ASF1b in AGS cells slowed tumor growth *in vivo*

**Figure 8:**
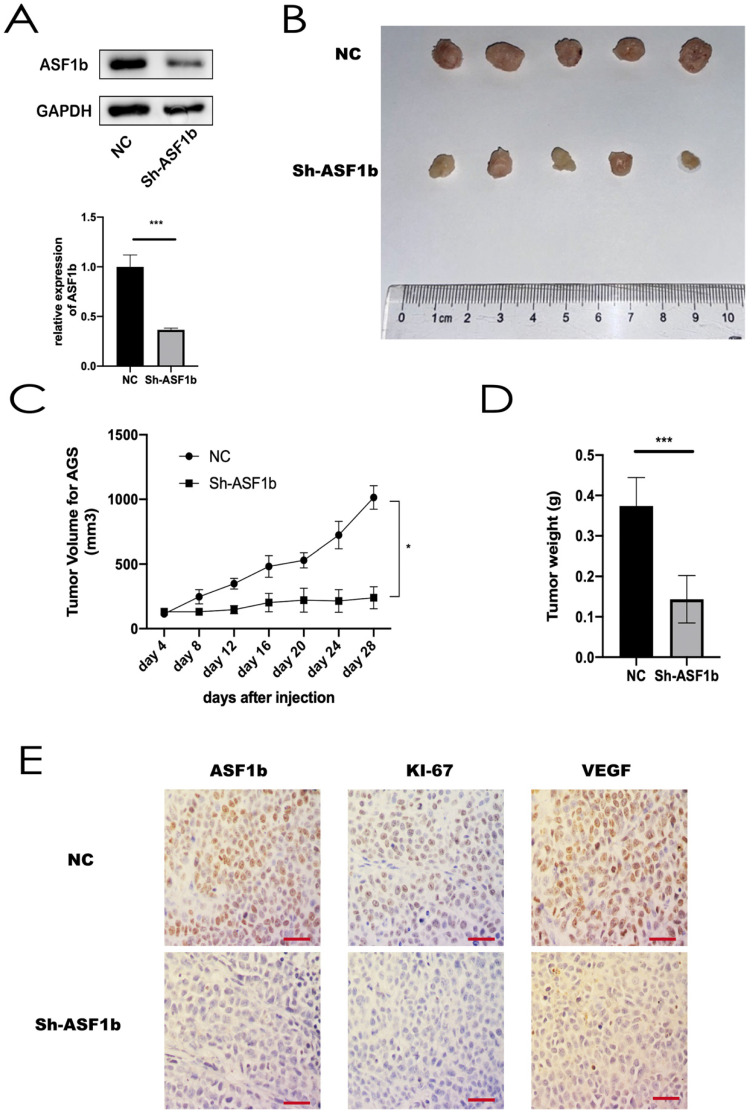
(A) Protein expression and mRNA level of ASF1b were efficiently inhibited by Sh-ASF1b in AGS. (B) Representative images of subcutaneous tumors in BALB/c nude mice injected with AGS cells transferred with stably Sh-ASF1b/NC. (C,D) Tumor volumes and final body weights of mice injected with AGS cells transferred with stably Sh-ASF1b/NC. (E) ASF1b, Ki-67 and VEGF staining with corresponding antibody in xenografted AGS tumors after ASF1b silencing or NC, Vascular endothelial growth factor (VEGF), scale bar = 10 μm.

To assess the effects of ASF1b on tumorigenic ability *in vivo*, AGS cells stably transfected with shRNA-NC and shRNA-ASF1b were subcutaneously injected into the left flanks of each BALB/c nude mice (4 weeks old), respectively (Figure 8A). There was no difference in body weight in the two groups. Tumor sizes were measured every four days. After 8 days, the measured data showed shRNA-ASF1b tumors grew more slowly than the shRNA-NC tumors (Figure 8B and 8C). Animals were sacrificed by cervical dislocation 28 days after inoculation. And the tumors were removed for subsequent experiments. Consistently, the tumors in the shRNA-ASF1b groups were weighed less than those of the shRNA-NC group (Figure 8D). By IHC staining of the paraffin sections of the tumors, it was found that the Ki-67 and VEGF expression in the NC group was higher than in the shRNA-ASF1b, and ASF1b in the NC group was higher than in the shRNA-ASF1b groups (Figure 8E). These *in vivo* results support that ASF1b improves tumor vascular function and promotes tumor growth in GC.

## Discussion

GC, a major cause of cancer-related deaths worldwide, represents one of the most common malignancy. Although some biomarkers, such as CEA, CA199, AFP, CA125, Her-2 and serum Helicobacter pylori antibodies 26, 27, have been reported as the prognostic value in gastric cancer patients, more progress in early diagnosis and treatment is urgently needed. Therefore, increased efforts to develop valid, reliable biomarkers in GC is the focus of extensive research. In this study, we investigated the role of ASF1b in predicting poor prognosis of GC. Firstly, we used a straightforward procedure to evaluate the expression feature and clinical relevance of ASF1b and found that exceptional high expression of ASF1b occurs in GC tumors. Then we confirmed its prognostic value by comprehensive analysis of cancer databases and our clinical samples (Figure 1). Moreover, we induced the knockdown and overexpression of ASF1b to explore its function and mechanism, which revealed that the disrupting the ASF1b not only suppressed cancer cell proliferation *in vitro* but also slowed the growth of GC cell-derived xenograft tumors *in vivo*. Our results indicated that ASF1b functions as an oncogene in promoting GC progression.

The advent of novel biomarkers with diagnostic and prognostic value has led to a paradigm shift in tumor classification. In recent years, researchers have achieved considerable success in categorizing GC into different molecular subtyping, with distinct clinical prognosis to guide targeted therapy. Interpretation of genomic data provided a theoretical basis for molecular classification of cancer and a more accurate diagnosis for GC patients 28, 29.

ASF1b has been shown as an oncogene to promote cervical cancer, breast cancer, cell renal cell carcinoma and prostate cancer 23, 30-32. Compared to NAT, increased expression of ASF1b was reported in various cancer, and its high expression was predictive of poor prognosis. Liu X et al. suggested that higher ASF1b levels was positively correlated with tumor size in cervical cancer 23. Corpet A reported that ASF1b was a proliferation marker of prognostic, whose upregulation remarkably associated with an increased occurrence of distant metastasis and a shorter OS 30. Moreover, ASF1b levels also identify the aggressivity of prostate cancer subtypes, with high tumor N stage and M stage 31. ASF1b, as one of the isoforms of ASF1 histone H3-H4 chaperone in mammals, acted as the key histone acceptor/donor in mitosis progression. The depletion of ASF1b possibly drove a consequence of impaired chromatin assembly during S-phase 22, 30. One particular physiological context highly associated with human cancer is proliferation.

Given these findings, we hypothesized that ASF1b is involved in the tumorigenesis of GC and progression. As is shown in Figure 5, our CCK-8 and colony formation assay indicated that ASF1b depletion suppressed proliferation, while opposite results were found in GC cells with ASF1b overexpression. Moreover, our KEGG and GO analyses suggested that ASF1b promoted cell proliferation through cell cycle control. Specifically, ASF1b was involved in organelle fission, nuclear division, and DNA replication, i.e., S-phase and M-phase. It has been reported that the accurate replication of genomic DNA is encoded during the S phase of the cell cycle 33. This cell cycle regulation was further confirmed by a series of flow cytometry experiments, which showed that ASF1b knockdown induced GC cells arrest in the G0/G1 phase (Figure 7). Previous studies identified that increasing the activity of CDK2, CDK4, and cyclin D1 can accelerate the transition of cells from G1 to S phase, and finally promote the proliferation of cancer cells 34, 35. In this study, we found that ASF1b expression positively correlated with the expression of CDK2, CDK4 and cyclin D1. However, CDK inhibitors P21 and P27 were negatively correlated with ASF1B expression. Consistent with known results, a switch like G1 to S phase entry is primarily driven by the feedback loop between CDK2/CDK4 and CDK inhibitor P21/P27 36. Using gain- and loss-of-function strategies, we further verified the changes of these cell cycle markers by qPCR. And similar results were observed as seen in Figure 6. More importantly, parallel results were observed in AGS cells with ASF1b-si *in vivo*. The expression of proliferation-associated protein (Ki-67), was significantly inhibited in ASF1b-si group. Our research highlighted cell cycle regulation, as one of the major mechanisms of cell growth, was highly related to the promotion of cancer by ASF1b.

To explore the potential underlying mechanism of ASF1b in invasion and migration of GC, we found ASF1b may be involved in PI3K/AKT/mTOR pathway in GC. PI3K/AKT/mTOR pathway activation serves a critical role in cell growth, proliferation and cell repair, and has been observed in many types of tumors 37, 38.A previous study demonstrated that activation of the PI3K/AKT/mTOR pathway accelerated the metastasis of gastric cancer 39. In addition, high expression of ASF1b expression was associated with increased incidence of breast cancer in progression and metastasis 30. Thus, the expression of PI3K, AKT, mTOR and their phosphorylation level was determined in our study in order to investigate the role of PI3K/AKT/mTOR pathway in the anti-tumor effect of ASF1b-si. In present study, we showed ASF1b regulated the phosphorylation of mTOR. At the same time, knockdown of ASF1b decreased the phosphorylation levels of PI3K and AKT in AGS and MGC803 cells (Figure 6E). Therefore, activation of the PI3K/AKT/mTOR pathway may be a potential mechanism underlying the carcinogenic effect of ASF1b in GC.

However, our study had some limitations. First, this was a single centre retrospective study which enrolled comparatively small sample size of patients with GC. Moreover, no ASF1b inhibitors had been reported, and the function of cell cycle signaling pathway during ASF1b upregulation required further investigation by corresponding inhibitors. In future, relevant genes of ASF1b needed be explored in transcription or translation. Interestingly and noteworthy, this was the first study systematically reported the role of ASF1b in GC. In our cohort, we investigated both the mRNA and IHC of ASF1b in GC samples. Additionally, the expression of ASF1b at different levels in GC cells was explored to further verify the conclusions revealed by the dataset. Lastly, the experiments *in vivo* rendered our research conclusions more reliable and trustworthy.

## Conclusions

In conclusion, high levels of ASF1b correlate with poor clinical outcome in GC patients. Functional and mechanistic studies suggested that ASF1b significantly induce GC cell proliferation and metastasis. In additional, ASF1b is involved in cell cycle and PI3K/AKT/mTOR pathways in GC cells. Therefore, this study defines ASF1b as a novel prognostic factor biomarker of clinical value. Future work should concentrate on leveraging these findings that highlight ASF1b as a potential therapeutic target for GC.

## Supplementary Material

Supplementary figures.

## Figures and Tables

**Table I:** Oligonucleotide sequences for this study.

Target	Sense (+) Antisense (-)	Sequence (5'-3')
**qPCR-Primer**		
ASF1b	+	CCAAGGTGTCGGTGCTGAA
	-	TCGAAGCTGATCTCGAACCG
CDK2	+	ACAAGCCAAGTTTCCCCAAGT
	-	TCCGCTTGTTAGGGTCGTAG
CDK4	+	GTGTATGGGGCCGTAGGAAC
	-	CCATAGGCACCGACACCAAT
CCND1	+	GAAGGAGACCATCCCCCTGA
(cyclin D1)	-	CAATGAAATCGTGCGGGGTC
CDKN1A	+	CCAGCATGACAGATTTCTACCAC
(P21)	-	GATGTAGAGCGGGCCTTTGA
CDKN1B	+	CGTCGGGGTCTGTGTCTTTT
(P27)	-	CTCCCGTTAGACACTCGCAC
GAPDH	+	CTTAGCACCCCTGGCCAAG
	-	GATGTTCTGGAGAGCCCCG
**siRNA, shRNA**		
ASF1b-si	+	CAGGCGGGAAUGUUAGUUATT
	-	UAACUAACAUUCCCGCCUGTT
shRNA-ASF1B	+	CACCGCCTGGAGTGGAAGATCATTTCAAGAGAATGATCTTCCACTCCAGGTTTTTTG
	-	GATCCAAAAAACCTGGAGTGGAAGATCATTCTCTTGAAATGATCTTCCACTCCAGGC

**Table II:** ASF1b with and clinicopathological features of 160 GC in ZJU.

Variable		ASF1B (n)		
		Low	High	P
Age	≥60	45	64	0.804^a^
	<60	20	31	
Gender	Male	47	66	0.699^a^
	Female	18	29	
Grade	G1/G2	18	11	**0.009^a^**
	G3	47	84	
T stage	T1/T2	7	6	0.381^b^
	T3/T4	58	89	
N stage	N0/N1	31	36	0.217^a^
	N2/N3	34	59	
M stage	M0	61	92	0.443^b^
	M1	4	3	
AJCC stage	I-II	23	16	**0.007^a^**
	III-IV	42	79	

^a^Chi-square test, ^b^Yates'continuity corrected chi-square.

## Abbreviations

GC): Gastric cancer
ASF1b): Anti-silencing function 1b
IARC): International Agency for Research on Cancer
NGS): Next-generation sequencing
MSI): microsatellite instability
HER2): Human epidermal growth factor receptor-2
GEPIA): Gene Expression Profiling Interactive Analysis
DEGs): differential expression genes
STAD): stomach adenocarcinomas
ASF1): anti-silencing function 1
qPCR): Quantitative PCR
NAT): normal adjacent tissues
TMA): tissue microarray
IHC): immunohistochemistry
AJCC 8th edition): American Joint Committee on Cancer 8th edition
OS): overall survival
DFS): disease-free survival
CCK-8): Cell Counting Kit-8
HR): Hazard rate
KM plotter): Kaplan-Meier plotter
EGA): European Genome-phenome Archive
GO): Gene Oncology
KEGG): Kyoto Encyclopedia of Genes and Genomes
BP): biological processes
MF): molecular function
CC): cellular component
LAML): Acute Myeloid Leukemia
TGCT): Testicular Germ Cell Tumors
VEGF): vascular endothelial growth factor
