# Supplementary material for: ASF1b is a novel prognostic predictor associated with cell cycle signaling pathway in gastric cancer
Source: J Cancer. 2022 Mar 28;13(6):1985–2000. doi: 10.7150/jca.69544 (PMC8990430; doi:10.7150/jca.69544)
Supplement: Supplementary file 1 — Supplementary figures. [file jcav13p1985s1.pdf]

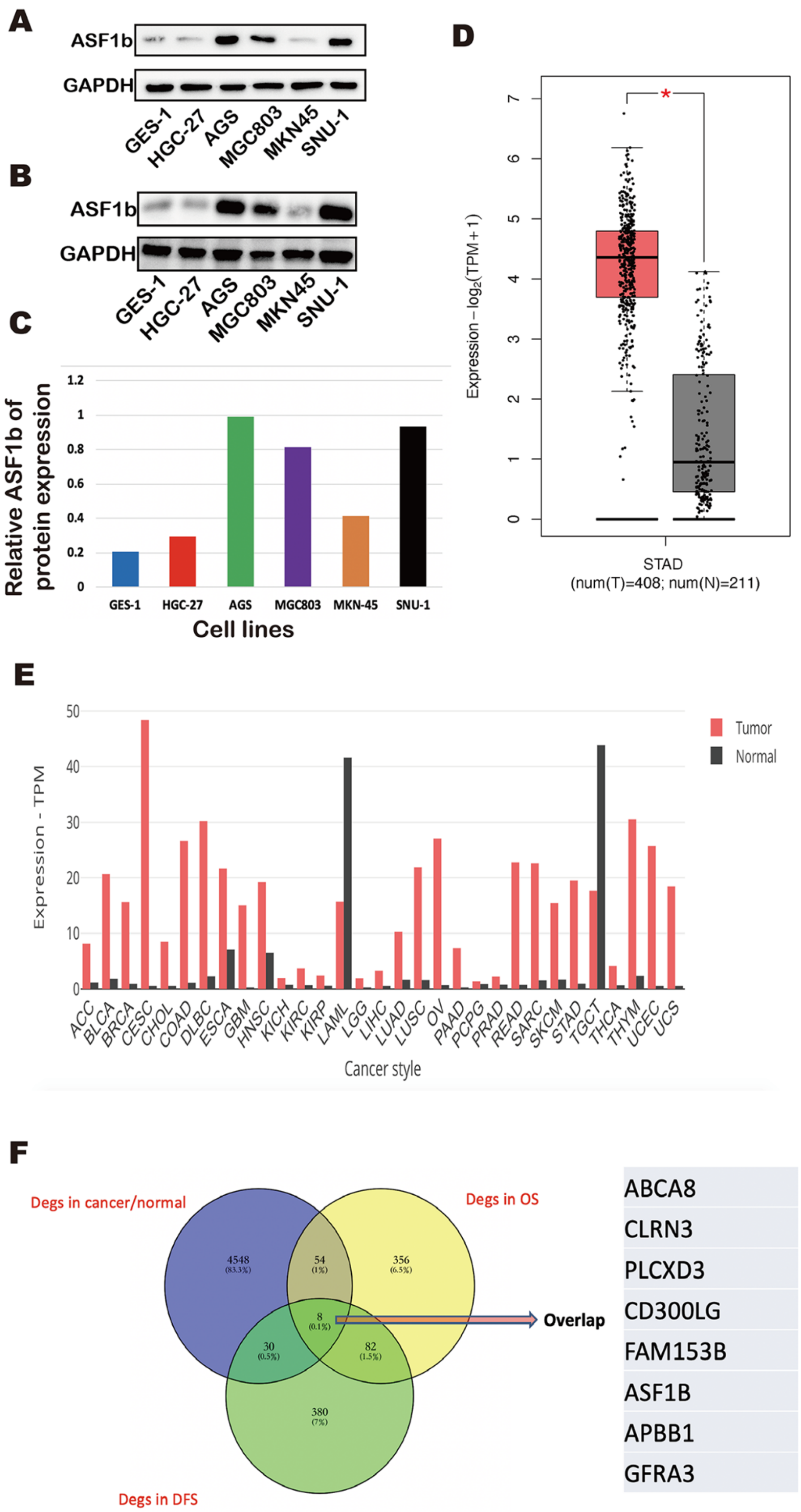

Figure S1

FiGURE S1. (A,B) Protein expression of ASF1b were repeated in six human GC cell

lines. (C) Mean gray levels of the three times protein bands were quantified by using the ImageJ program. (D) The intersection of the three gene sets to obtain 8 genes based on GEPIA database.

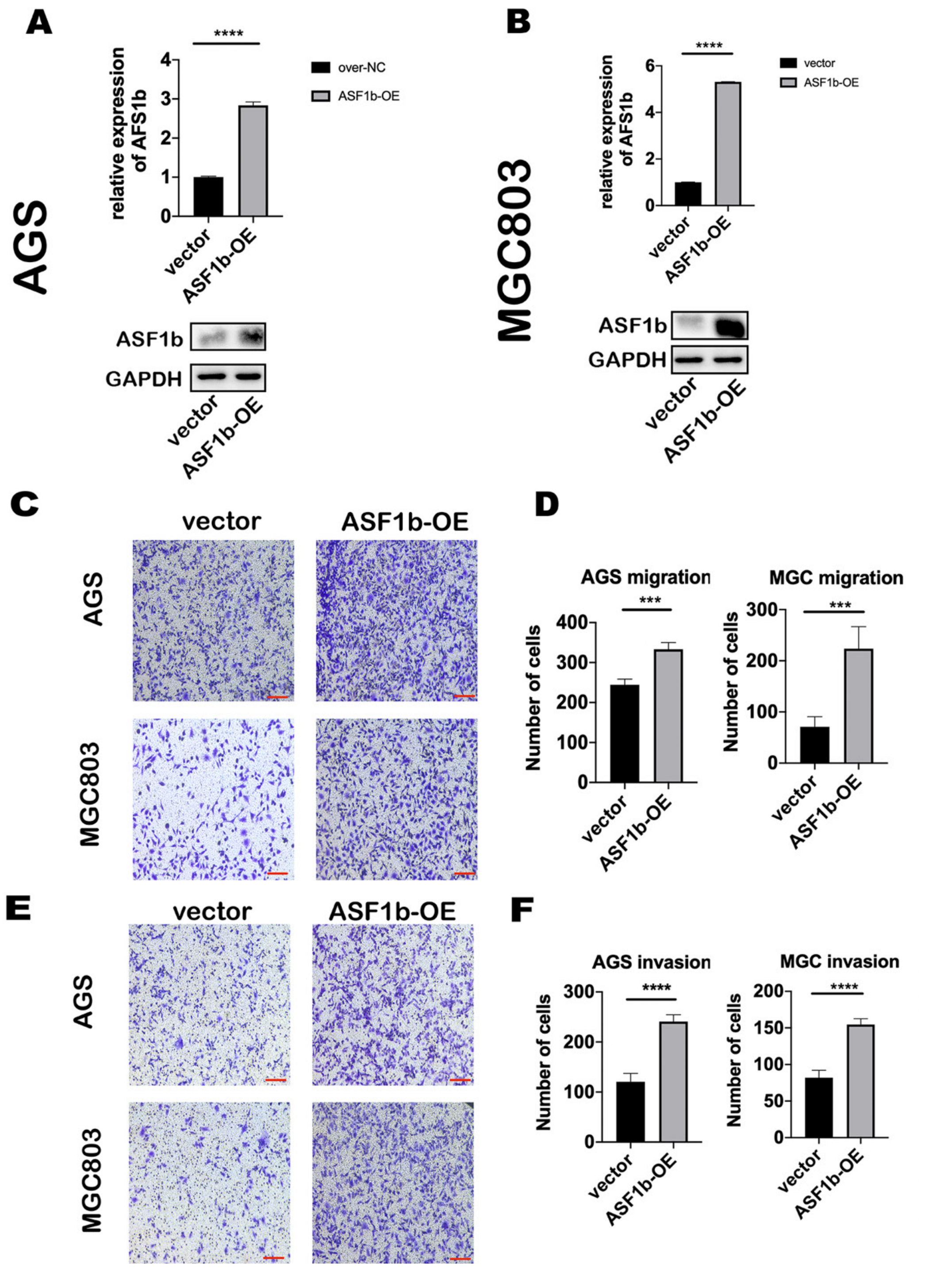

Figure S2

FiGURE S2. (A,B) MRNA and protein expression level of ASF1b were efficiently unregulated by ASF1b-OE in AGS and MGC803 cells. (C,D) Migration assay was performed in AGS and MGC803 cells transfected with ASF1b-OE/vector (scale bar = 10 μm, ***P < 0.001). (E,F) Invasion assay was performed in AGS and MGC803 cells

transfected with SF1b-OE/vector (scale bar = 10 μm, ****P < 0.0001).
